# Managing non-proportionality of hazards (PH) within TNT: a randomised phase III trial of carboplatin compared to docetaxel for patients with metastatic or recurrent locally advanced triple negative (TN) or brca1/2 breast cancer (BC)

**DOI:** 10.1186/1745-6215-16-S2-P150

**Published:** 2015-11-16

**Authors:** Holly Tovey, Judith Bliss, Andrew Tutt, James Morden, Katy Jarman, Sue Martin, Sarah Kernaghan, Christy Toms, Lucy Kilburn

**Affiliations:** 1 Institute of Cancer Research Clinical Trials & Statistics Unit (ICR-CTSU), London, UK; 2 King's College London School of Medicine, London, UK

## Introduction

TNT is a trial comparing carboplatin with docetaxel for advanced TN or BRCA1/2 BC patients. TNBC is heterogeneous; median progression-free survival (PFS) is short but some patients have prolonged PFS following treatment. BRCA1/2 patients were hypothesised to respond better to carboplatin. This interaction, with the heterogeneous population, was expected to cause a failure of PH assumptions required for Cox-PH analysis. Therefore, TNT required alternative analysis methods.

## Methods

376 patients were randomised (1:1) to carboplatin or docetaxel for 6-8 cycles or until progression. Primary endpoint was objective response; PFS was a secondary endpoint. Restricted mean survival (RMS) was used to deal with non-PH. A cut-off of 15months was used. Treatment groups were compared using a t-test. A Cox-PH landmark analysis (LA) splitting data at 3, 6 and 9months and a time-dependent Cox model were also applied.

## Results

Kaplan-Meier curves crossed and the PH assumption didn't hold (p=0.02). RMS indicated no difference in PFS between groups; difference=0.4 months (-1.1-0.3); p=0.29. Within each period of the LA, PH assumptions held. Between 0-3months PFS is better for patients on docetaxel (HR=1.73, p=0.001) however for patients with PFS> 6months this reversed (HR=0.63, p=0.05). Beyond 9months there is no significant difference (HR=0.82, p=0.56). A time-dependent Cox model resulted in the same HRs as the LA.

## Discussion

Lack of PH is a common issue in metastatic cancer trials. Often this is ignored and standard Cox regression applied or modelling avoided. TNT has shown that RMS is a suitable alternative for comparing groups in the presence of non-PH.

